# The Development of a Resting Metabolic Rate Prediction Equation for Professional Male Rugby Union Players

**DOI:** 10.3390/nu16020271

**Published:** 2024-01-16

**Authors:** Logan Posthumus, Matthew Driller, Paul Winwood, Nicholas Gill

**Affiliations:** 1 Te Huataki Waiora School of Health, The University of Waikato, Hamilton 3216, New Zealand; logan.posthumus@nzrugby.co.nz; 2 New Zealand Rugby, Wellington 6011, New Zealand; 3 Faculty of Health, Education and Environment, Toi Ohomai Institute of Technology, Tauranga 3112, New Zealand; paul.winwood@toiohomai.ac.nz; 4 Sport, Performance, and Nutrition Research Group, School of Allied Health, Human Services and Sport, Melbourne 3086, Australia; m.driller@latrobe.edu.au; 5 Department of Sport and Recreation, Sports Performance Research Institute New Zealand, Auckland University of Technology, Auckland 0627, New Zealand

**Keywords:** energy demands, body composition, indirect calorimetry, resting energy expenditure

## Abstract

Determining resting metabolic rate (RMR) is an important aspect when calculating energy requirements for professional rugby union players. Prediction equations are often used for convenience to estimate RMR. However, the accuracy of current prediction equations for professional rugby union players remains unclear. The aims of this study were to examine the RMR of professional male rugby union players compared to nine commonly used prediction equations and develop and validate RMR prediction equations specific to professional male rugby union players. One hundred and eight players (body mass (BM) = 102.9 ± 13.3 kg; fat-free mass (FFM) = 84.8 ± 10.2 kg) undertook Dual-energy X-ray Absorptiometry scans to assess body composition and indirect calorimetry to determine RMR. Mean RMR values of 2585 ± 176 kcal∙day^−1^ were observed among the group with forwards (2706 ± 94 kcal·day^−1^), demonstrating significantly (*p* < 0.01; *d* = 1.93) higher RMR compared to backs (2465 ± 156 kcal·day^−1^), which appeared to be due to their higher BM and FFM measures. Compared to the measured RMR for the group, seven of the nine commonly used prediction equations significantly (*p* < 0.05) under-estimated RMR (−104–346 kcal·day^−1^), and one equation significantly (*p* < 0.01) over-estimated RMR (192 kcal·day^−1^). This led to the development of a new prediction equation using stepwise linear regression, which determined that the strongest predictor of RMR for this group was FFM alone (*R*^2^ = 0.70; SEE = 96.65), followed by BM alone (*R*^2^ = 0.65; SEE = 104.97). Measuring RMR within a group of professional male rugby union players is important, as current prediction equations may under- or over-estimate RMR. If direct measures of RMR cannot be obtained, we propose the newly developed prediction equations be used to estimate RMR within professional male rugby union players. Otherwise, developing team- and/or group-specific prediction equations is encouraged.

## 1. Introduction

Over the course of a typical seven-day competition week, professional male rugby union (RU) players have been observed to train up to four days per week, comprising three resistance-training sessions, five field-training sessions, and if selected, a match on game day [1,2]. To support the training and game demands of RU players, it is important to optimise energy intake in relation to energy expenditure to meet individual player needs and enhance performance and recovery [3]. A squad of RU players present a diverse range of physical and fitness characteristics [4] and therefore possess unique individual energy requirements.

Often, players may need to achieve a higher body mass (in the desired form of lean muscle mass), which is typically achieved by being in an energy surplus for an extended period of time alongside appropriate training and macronutrient composition [5,6]. In contrast, players may need to acquire a lower body mass (BM) and/or lower body fat mass (FM) while preserving as much lean muscle mass as possible, which is often achieved by being in an energy deficit for an extended period of time [7] and also combined with specific training and macronutrient composition [8,9]. Players considered to be at an appropriate/optimal BM, body composition, and fitness level according to the athlete and/or coaching staff, may simply be aiming to meet energy requirements to optimise performance, recovery, and overall well-being [3,10].

Measuring or predicting resting metabolic rate (RMR) is often the first step in determining energy intake in general and in athletic populations because RMR substantially contributes (~50–60%) to total daily energy expenditure [10]. By definition, RMR is the amount of energy required to satisfy physiological processes in a rested, fasted, and inactive condition [11]. Direct measurement of RMR using indirect calorimetry is desired to individualise energy requirements with the most certainty [10]. However, this process requires expensive equipment, trained personnel, and time. The burden on athletes and coaches is also considerable due to the athlete needing to be in a well-rested and overnight-fasted state to collect accurate and reliable measures [12,13]. Therefore, practitioners often seek RMR prediction equations to provide an estimate of the athletes’ RMR using physical characteristic variables, such as age, height, BM, and fat-free mass (FFM) [10].

Prediction equations based on BM have been used for well over a century with many more being developed over recent years to save time and money for both the practitioner and the athlete [14]. However, many of these prediction equations have been generated using non-athletes or athletes with substantially lower stature, body mass, and in particular, FFM than those of professional RU players [15]. Previous studies among rugby players have demonstrated that current prediction equations can considerably misreport RMR compared to direct measures [15,16,17]. Other sports involving similarly large athletes [14,18] have also suggested that prediction equations that are not sport-specific may not be appropriate for athletes with substantially more FFM and may therefore under-estimate RMR. This could result in deficiencies in energy, which are associated with performance decrements, losses in FFM, and increased susceptibility to illness and/or injury [19]. Consequently, sport-specific prediction equations are strongly encouraged to better estimate energy requirements among specific athletes in their respective sports [18,20,21,22].

A population-specific prediction equation is likely important for RU players due to the large body and FFM these athletes possess and the substantial energy requirements these athletes demonstrate [3]. MacKenzie-Shalders et al. [15] presented a prediction equation among a small sample (*n* = 18) of young developing RU players. Popular and well-used prediction equations have used sample sizes of 50 to over 100 participants [10,23]. Using a large sample size of professional RU players across a range of positions and ages is desired to accurately predict RMR and inform energy requirements within this population group.

Therefore, the aims of this study were to (a) measure the RMR of professional male RU players and compare them to currently established prediction equations, and (b) develop and validate a population-specific prediction equation for professional male RU players if current prediction equations are not suitable. It was hypothesised that the currently established prediction equations would significantly under-estimate RMR in professional RU players due to the larger BM and FFM associated with the sporting demands of these athletes.

## 2. Materials and Methods

### 2.1. Study Design

A cross-sectional study design was utilised to assess the body composition and RMR of professional male RU players under standardised conditions; >8 h fast and >12 h after exercise [10,12,13] between 05:00 and 09:00 h. Body composition and RMR testing were assessed a week apart from each other during the pre-season phase. Measured RMR was then compared to commonly used prediction equations, followed by the development and internal validation of a new population-specific prediction equation for RMR.

### 2.2. Participants

One hundred and eight male professional RU players participated in this study. Players were from New Zealand teams competing in the Super Rugby Pacific Championship (*n* = 64), and are classified as elite worldclass for team sports as they are from Super Rugby and International levels [24]. Players were also from the New Zealand National Provincial Championship (*n* = 44), and are classified as highly trained/national level for team sports [24]. Players were categorised according to their primary playing positions, comprising 54 forwards and 54 backs. All participants provided informed consent, and ethical approval was obtained from the University of Waikato Human Research Ethics Committee (HREC2019#04).

### 2.3. Anthropometric and Body Composition Assessment

Players were instructed to arrive at the laboratory in a rested (>12 h following exercise or strenuous physical activity) and fasted (>8 h) state, with all jewellery removed and wearing underwear and/or tight-fitting compression clothing free from zips, studs, metal, and reflective objects [25]. Players were measured for height using a fixed-to-wall stadiometer (SECA Scales, Hamburg, Germany) measuring to the nearest 0.5 cm, and BM was measured using a calibrated set of scales (SECA Scales, Hamburg, Germany) measuring to the nearest 0.1 kg.

Each player then underwent a whole-body fan-beam Dual-energy X-ray Absorptiometry (DXA) scan (Hologic QDR Series, Discovery A, Bedford, MA, USA) following methods previously described [4] to provide FFM, FM, and percent body fat (Fat%). All scans were performed and analysed by the same trained operator. Body composition measures were reported as whole-body, which included the head.

### 2.4. RMR Assessment

One week following the anthropometric and body composition assessment, players were again instructed to arrive at the laboratory in a rested (>12 h) and fasted (>8 h) state [10,12,13]. Players underwent a 20 min rest period in a reclined, seated position wearing a face mask (7450 Series Silicone V2, Hans Rudolph, KS, USA), which allowed the players to familiarise themselves and settle their breathing before the beginning of a 20 min indirect calorimetry RMR assessment. The assessment took place in a dimly lit and temperature-controlled room (21–23 °C), with players lying quietly in a supine position [10,12,13].

Expired gas was analysed using a breath-by-breath gas analyser (MetaLyzer 3B-R3, Cortex Biophysik GmbH, Leipzig, Germany), which has demonstrated good accuracy for respiratory parameters and total energy expenditure [26]. The gas analyser was calibrated as per the manufacturer’s guidelines using two known concentrations of each gas (ambient and 15% O_2_ and ambient and 5% CO_2_), daily barometric pressure, and a 3 L volume syringe. The player’s face mask was then connected to a gas analyser for high-resolution, breath-by-breath analysis. The first five minutes of the RMR test were discarded [12]. A 5 min steady state interval was achieved during the remaining 15 min, providing an accurate assessment of RMR with a coefficient of variation < 10% [13]. The Weir equation [27] was used to calculate RMR.

### 2.5. Current Commonly Used Prediction Equations for RMR

Resting metabolic rate was estimated for each player using nine different prediction equations from six studies [14,15,23,28,29,30] (Table 1). These equations were selected as they have been routinely used to predict RMR in rugby players [15,16,17] and have been shown to under- or over-estimate RMR in this population. Moreover, these prediction equations were developed using a similar sample size to that used in the present study.

### 2.6. Statistical Analyses

All data were analysed using the Statistical Package for the Social Sciences (SPSS, v28, Chicago, IL, USA). Data were checked for normality using the Shapiro–Wilk test. Differences in anthropometrics, body composition, and RMR between positions and levels were analysed using independent *t* tests. Relationships between physical characteristics and measured RMR were determined using Pearson correlation coefficients (*r*) with the following thresholds: *trivial* (<0.09), *small* (0.10–0.29), *moderate* (0.30–0.49), *large* (0.50–0.69), *very large* (0.70–0.89), or *nearly perfect* (0.90–0.99) [31]. Comparisons between the measured RMR value and predicted values were analysed using repeated-measures analysis of variance (ANOVA) with Bonferroni post hoc. Effect sizes were calculated using the Cohen’s *d* method with the following thresholds: *d* = *trivial* < 0.19, *small* 0.20–0.39, *moderate* 0.40–0.59, *large* 0.60–0.79, and *very large* > 0.80 [32].

Stepwise multiple regression analysis was carried out using relevant variables (age, height, BM, FFM, and FM) to develop a new RMR prediction equation for professional male RU players. The significantly strongest predictors of RMR were both FFM and BM alone, resulting in an FFM-based and BM-based RMR prediction equation. The root mean squared error (RMSE) was calculated as the expected absolute deviation (kcal·day^−1^) of the predicted RMR from the measured RMR. Bland–Altman plots were used to identify the 95% limits of agreement between the measured RMR and the predicted RMR. Internal validation of the newly developed prediction equations were carried out using *k*-fold cross-validation (*k* = 10) where the data set was randomly separated into 10 folds. Nine folds were used to develop/train the prediction equation and was then tested on the 10th fold. This process was repeated until all folds were used in both the development and testing of each model [33]. The average of the mean difference, mean absolute percentage error (MAPE), RMSE, *r*, *R*^2^, and *p* values for the 10 folds were reported [34]. The statistical significance was set at *p* < 0.05.

## 3. Results

### 3.1. Demographics, Body Composition, and RMR

Demographics, body composition, and resting metabolic rate are presented in Table 2. Forwards demonstrated significantly greater height (*p* < 0.01; *d* = 1.06), BM (*p* < 0.01; *d* = 2.34), FFM (*p* < 0.01; *d* = 2.04), FM (*p* < 0.01; *d* = 1.64), Fat% (*p* < 0.01; *d* = 0.78), and RMR (*p* < 0.01; *d* = 1.93) compared to backs. No significant differences were observed in RMR between Super Rugby and National Provincial Union forwards (2709 ± 101 versus 2699 ± 75 kcal∙day^−1^, respectively, *p* = 0.74; *d* = 0.10) and backs (2499 ± 128 versus 2437 ± 172 kcal∙day^−1^, respectively, *p* = 0.15; *d* = 0.40).

### 3.2. Relationship between Predictor Variables and RMR

Correlation coefficients between potential predictor variables and RMR can be observed in Table 3. The strongest correlations observed were *very large* between FFM and RMR (*r* = 0.84; *p* < 0.01) and BM and RMR (*r* = 0.81; *p* < 0.01). A *nearly perfect* relationship (*r* = 0.96; *p* < 0.01) was also observed between FFM and BM among this group.

### 3.3. Measured RMR Compared to Current and Newly Developed Prediction Equations

Comparisons between measured RMR and predicted RMR values can be observed in Table 4. There was a significant difference between measured and predicted RMR (*F*(2.77, 295.88) = 350.84, *p* < 0.01). Of the nine commonly used prediction equations from six studies, seven significantly (*p* < 0.01) under-estimated RMR [14,15,23,28,29] compared to measured values, except for Tinsley^BM^ [14], which only marginally under-estimated RMR. However, Jagim [30] significantly (*p* < 0.01) over-estimated RMR compared to measured RMR values.

**Figure 1:**
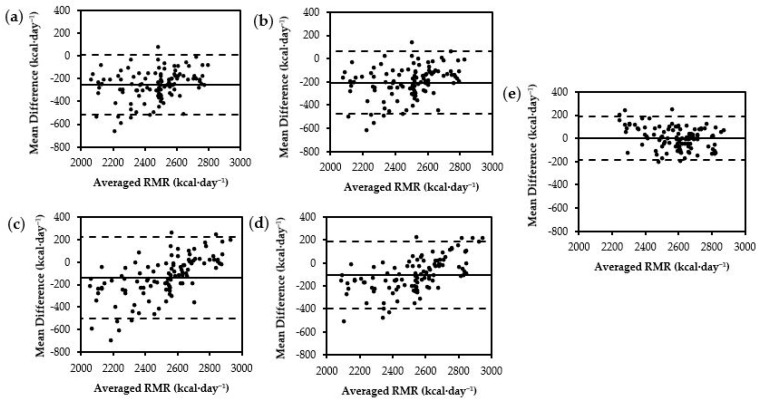
Bland–Altman plots for FFM equations: (**a**) Cunningham, (**b**) ten Haaf FFM, (**c**) Mackenzie-Shalders LBM, (**d**) Tinsley FFM, (**e**) Posthumus FFM. The middle, solid, horizontal line represents the constant error. The lower and upper dashed horizontal lines represent the 95% limits of agreement.

**Figure 2:**
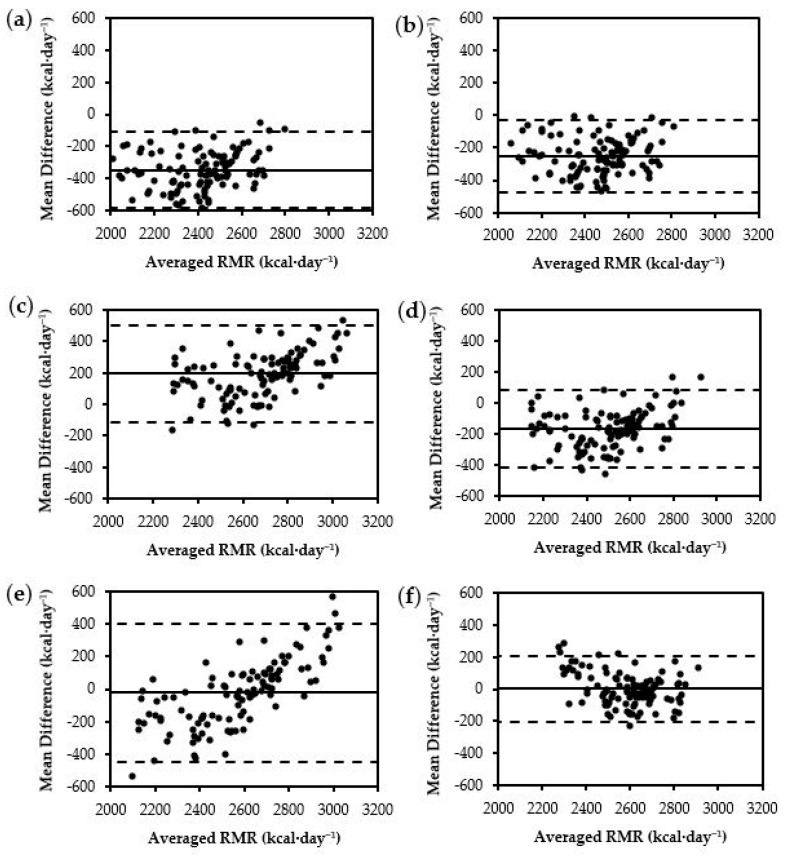
Bland–Altman plots for BM equations: (**a**) Harris–Benedict, (**b**) ten Haaf BM, (**c**) Jagim, (**d**) Mackenzie-Shalders BM, (**e**) Tinsley BM, (**f**) Posthumus BM. The middle, solid, horizontal line represents the constant error. The lower and upper dashed horizontal lines represent the 95% limits of agreement.

Both newly developed prediction equations from the current study demonstrated the lowest RMSE (FFM = 96 kcal·day^−1^, BM = 104 kcal·day^−1^) compared to all commonly used equations. Out of the nine selected commonly used prediction equations, the Tinsley^FFM^ equation demonstrated the next lowest RMSE (182 kcal·day^−1^). Upon visual inspection of the limits of agreement using Bland–Altman plots, the newly developed prediction equations demonstrated the lowest fixed and proportional bias compared to the commonly used prediction equations (Figure 1e and Figure 2f).

### 3.4. Newly Developed Prediction Equation Compared to Measured RMR

**Figure 3:**
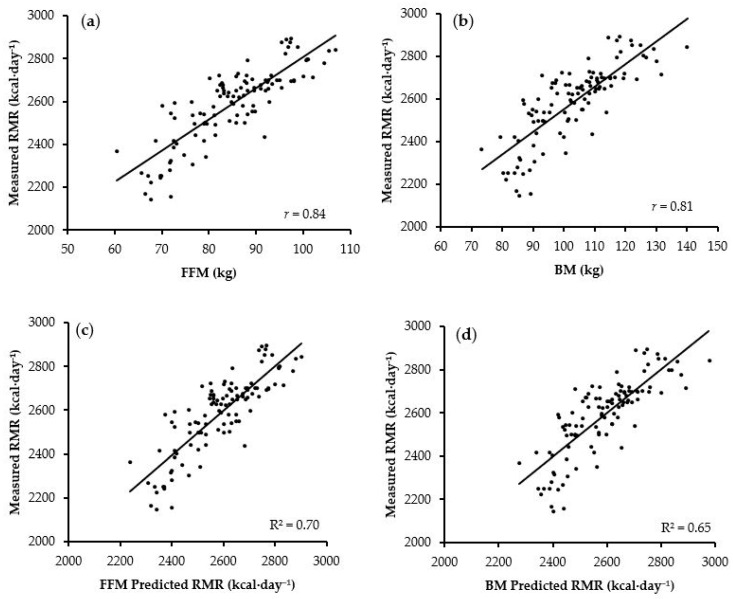
Relationship between (**a**) fat-free mass (FFM), (**b**) body mass (BM), and measured resting metabolic rate (RMR). The newly developed (**c**) FFM and (**d**) BM prediction equations plotted against measured RMR.

Stepwise multiple regression revealed that FFM (*R*^2^ = 0.70; Standard Error of Estimate (SEE) = 96.65) was the strongest significant predictor of RMR (*p* < 0.05), accounting for 70% of the variation in RMR, and was the only predictor variable included in the novel prediction equation. All other variables (age, height, BM, and FM) were rejected as they did not significantly improve the fit of the model. The predicted RMR values are plotted against measured RMR values (Figure 3c), and the relationship between measured RMR and FFM is plotted (Figure 3a).

Fat-Free Mass RMR Prediction Equation (kcal·day^−1^) = 1351.74 + (14.53 × FFM in kg) (1)

A second prediction equation using BM was derived due to the ease of assessment compared to the need for specialised equipment to obtain accurate FFM measures. A second stepwise multiple regression was carried out with age, height, and BM entered as predictor variables, with BM being the single best predictor (*R*^2^ = 0.65; SEE = 104.97; *p* < 0.05), accounting for 65% of the variation in RMR. Both age and height were rejected as they did not significantly improve the fit of the model. The predicted RMR values are plotted against measured RMR values (Figure 3d), and the relationship between measured RMR and BM are plotted (Figure 3b).

Body Mass RMR Prediction Equation (kcal·day^−1^) = 1489.93 + (10.63 × BM in kg) (2)

### 3.5. Internal Validation of the Newly Developed Prediction Equations

Following *k*-fold (*k* = 10) cross-validation, no significant differences were present between measured RMR and the newly developed FFM (*p* = 0.44) and BM (*p* = 0.42) prediction equations. *Very large* relationships were observed between measured RMR and the prediction equations based on FFM (*r* = 0.82) and BM (*r* = 0.82). The FFM-based prediction equation demonstrated marginally better MAPE (3.2 ± 2.4% vs. 3.4 ± 2.7%), *R*^2^ (0.69 vs. 0.68), and RMSE (98 vs. 105 kcal·day^−1^) than the BM prediction equation when compared to measured RMR.

Comparing the results from the validation data set with the whole data set used to develop the prediction equations, *R*^2^ only decreased by 0.01 for the FFM-based equation (R^2^ = 0.69 vs. 0.70). However, the BM-based equation demonstrated an increase in R^2^ by 0.03 (R^2^ = 0.68 vs. 0.65). The RMSE was also similar between the validation and whole group data for FFM (RMSE = 98 vs. 97 kcal·day^−1^) and BM (RMSE = 104 vs. 105 kcal·day^−1^).

## 4. Discussion

The current study measured the RMR of professional male RU players using indirect calorimetry and compared them to commonly used prediction equations [14,23,28,29,30] and one RU-specific prediction equation [15]. Our findings support our initial hypothesis where the majority of the selected current prediction equations significantly under-estimated [15,23,28,29] RMR in our group of professional RU players. The existing prediction equations were deemed inappropriate for this population; therefore, stepwise linear regression was carried out to develop a new, population-specific prediction equation for professional male RU players. The development of an FFM-based prediction equation was implemented due to FFM generating the significantly strongest model on its own by accounting for 70% of the variation in RMR. Due to the challenges of obtaining accurate and user-friendly measures of FFM, a BM-based prediction equation was also developed to allow RMR to be calculated conveniently for players and practitioners, with BM still presenting a strong model, accounting for 65% of the variation in RMR.

Our study observed a mean RMR of 2585 ± 176 kcal·day^−1^, which was higher than baseline measures observed in 22 English premiership RU players (2318 ± 182 kcal·day^−1^) [35] and 18 Australian young developing RU players (2389 ± 263 kcal·day^−1^) [15], but was similar to RMR measures observed one day following an English premiership RU game (2544 ± 397 kcal·day^−1^) [35]. We also reported higher RMR measures compared with 26 Under-16 to Under-24 RU (2123 ± 269 kcal·day^−1^) and Rugby League (2366 ± 296 kcal·day^−1^) players [17]. However, higher RMR measures were observed in young developing English Rugby League players (~2655 ± 516 kcal·day^−1^) [36,37] compared to our study.

Interestingly, player RMR generally increased linearly alongside FFM and BM, which was similar to observations reported by Costello et al. [37] in which the heaviest players presented the highest RMR. In contrast, Morehen et al. [16] reported that players with the lowest body mass and lean mass presented the highest RMR. In our study, we observed that although players could present a relatively high RMR with a relatively low body mass and/or FFM, typically as a player’s body mass and FFM increased, so did RMR. Given the larger sample size observed within our study, a linear trend was more present. Provided how much FFM has been shown to contribute to individual variations in RMR (25–70%) [38], this was expected. We also observed *nearly perfect* relationships between FFM and BM, which may explain why BM is also such a strong predictor for RMR in this group. Generally, as professional RU players grow larger, they demonstrate greater amounts of FFM with minimal increases in FM [4,39]. Meanwhile, when increases in BM are observed among the general population or lower playing levels, substantially more FM and less FFM is generally observed [10,39]. Therefore, in non-professional RU players, general prediction equations may be more accurate due to lesser amounts of FFM being present [4,39]. However, further studies are required to determine this.

After examining differences between positions within this study, we observed significantly higher RMR values among forwards (2706 ± 94 kcal·day^−1^) compared to backs (2465 ± 156 kcal·day^−1^). However, forwards also demonstrated significantly greater amounts of both FFM (92.0 ± 6.6 kg vs. 77.6 ± 7.6 kg) and BM (113.0 ± 9.2 kg vs. 92.8 ± 8.1 kg) compared to backs. Although these differences were observed between positions, when a stepwise linear regression was run separately among forwards and backs, the strength and significance of the prediction equation was not improved further. If position were included in the final model, this would have added another categorical step in the process by having to designate position which may not reflect what is being measured. This is because some RU backs, such as midfielders and outside backs, could be a similar size to or larger than a loose forward. Therefore, a position-specific prediction equation is likely not appropriate within this group, given increases in FFM and BM are predominantly linear (*r* = 0.96) across the group and account for a considerable amount of variation in RMR (FFM = 70% and BM = 65%).

When comparing the measured RMR within this study to current prediction equations, the Tinsley ^BM^ [14] equation demonstrated the smallest mean difference compared to the other equations used, but it demonstrated the largest variation in regards to standard deviation. The Tinsley ^FFM^ [14] and Mackenzie-Shalders ^LBM^ [15] equations were significantly lower than measured RMR, but given only *small* to *moderate* differences were observed, respectively, these equations were the next closest to predicting RMR in this group of RU players. It is likely that these two prediction equations [14,15] are more similar to the measured RMRs within our study because the participants had substantially greater amounts of FFM compared to the non-sport-specific prediction equations [28,29]. However, the Jagim [30] male prediction equation was also developed using heavier athletes (93.7 ± 16.3 kg) with considerable amounts of FFM (77.3 ± 8.1 kg) compared to older prediction equations [23,28,29], yet when using this equation within our group, RMR was significantly over-estimated (2778 ± 259 kcal·day^−1^) compared to our measured values (2584 ± 176 kcal·day^−1^). This may partly be due to the greater BM (102.9 ± 13.3 kg vs. 93.7 ± 16.3 kg) and FFM (84.8 ± 10.2 kg vs. 77.3 ± 8.1 kg) observed within our study compared to Jagim [30]. Additionally, we observed *very large* correlations between FFM and RMR (*r* = 0.84) and a *nearly perfect* relationship between FFM and BM (*r* = 0.96) compared to *moderate* correlations (*r* = 0.45) and *large* correlations (*r* = 0.69) observed by Jagim [30], respectively. Suggesting that, as BM increased within our group, RMR did not necessarily increase unless FFM was associated with the increase in BM, which could be another reason for the over-estimation by Jagim [30] and further justifies the importance of population-specific prediction equations.

It is also important to note that the non-team-sport-specific prediction equations [23,28,29] may have under-estimated RMR in our group due to our players likely being exposed to substantially greater training and sport-specific activities around RMR measurements compared to less active individuals. Aerobic exercise has demonstrated non-significant increases in RMR and resistance training has demonstrated significantly increased RMR measures [38]. Furthermore, the sport-specific activities of RU players involve high-intensity efforts (e.g., rapid accelerations and decelerations, high-speed running, and jumping), collisions (e.g., tackling, ball carrying, and colliding at breakdowns/rucks), and maximal-effort static exertions (scrumming and wrestling players in contact and at breakdowns/rucks) [40,41]. These sporting activities observed in rugby have been shown to elevate levels of RMR [35,42] and total daily energy expenditure [36].

Although every effort was carried out to ensure best-practice collection of RMR under stable conditions [12,13], RU players may possess an elevated RMR due to training and sport-specific demands. This further promotes the need for assessing RMR within the specific population group or developing sport-specific prediction equations to determine RMR. Future studies could assess the RMR of professional RU players in the off-season period to determine if RMR levels change due to fewer sporting and training demands. However, the practical application of assessing or determining RMR in athletes is to optimise energy requirements for their specific goal, which makes collecting RMR in the pre- and in-season phases important. Furthermore, when examining RMR, it is also important to take note of which device is used to directly assess RMR, as differences in mean values between measuring devices have been reported [14] and may also lead to differences observed between prediction equations and measures.

## 5. Conclusions

The results of this study demonstrate the importance of measuring RMR within athletes and in particular, professional male RU players as RMR can vary greatly between individuals and may be substantially under- or over-estimated using prediction equations that were not developed for this population group. This could lead to inaccuracies and inefficiencies when determining energy requirements for individual players’ nutrition goals and plans. Collecting or determining a more accurate measure of RMR may reduce the amount of initial energy adjustments required by the nutritionist and therefore lead to positive results sooner. If direct measures of RMR are difficult to obtain due to time or accessibility, the prediction equations developed within this study may be suitable for professional male RU players. If devices such as DXA are available to assess FFM, then the FFM-based equation should be used. However, if FFM is inconvenient to acquire due to limited access, the need for trained technicians, high cost, and limited time, then the BM-based equation should be used. Otherwise, developing team- or group-specific prediction equations is recommended.

## Figures and Tables

**Table 1:** Resting metabolic rate prediction equations.

Study	Sample	RMR Prediction Equation (kcal·day^−1^)
***Commonly Used Equations***		
Harris–Benedict [28]	M = 136 F = 103	M: 66.47 + (13.75 × BM) + (5.00 × H) − (6.75 − age)
Cunningham [29]	M = 120 F = 103	500 + (22 × FFM)
ten Haaf ^FFM^ [23]	M = 53 F = 37	484.26 + (22.77 × FFM)
ten Haaf ^BM^ [23]	M = 53 F = 37	29.28 + (11.94 × BM) + (5.88 × H) − (8.13 × age) + (191.03 × sex (M = 1, F = 0))
Jagim [30]	M = 68	M: 775.33 + (19.46 × BM)
Tinsley ^FFM^ [14]	M = 17 F = 10	284 + (25.9 × FFM)
Tinsley ^BM^ [14]	M = 17 F = 10	10 + (24.7 × BM)
***RU Specific Equations***		
Mackenzie-Shalders ^LBM^ [15]	M = 18	24.56 − (29.71 × FFM)
Mackenzie-Shalders ^BM^ [15]	M = 18	775.32 + (15.95 × BM)
***Newly Developed Equations***		
Posthumus (FFM equation)	M = 108	M: 1351.74 + (14.53 × FFM in kg)
Posthumus (BM equation)	M = 108	M: 1489.93 + (10.63 × BM in kg)

RMR = resting metabolic rate, M = male, F = female, BM = body mass, H = height, FFM = fat-free mass, RU = rugby union, LBM = lean body mass. Units for these equations are as follows: BM = kg, H = cm, age = years, FFM = kg, LBM = kg.

**Table 2:** Demographics, body composition, and resting metabolic rate.

	All Players (*n* = 108)	Forwards (*n* = 54)	Backs (*n* = 54)
Age (y)	25.7 ± 4.1	26.1 ± 3.7	25.3 ± 4.4
Height (cm)	185.8 ± 6.8	189.0 ± 6.3 *	182.6 ± 5.7
BM (kg)	102.9 ± 13.3	113.0 ± 9.2 *	92.8 ± 8.1
FFM (kg)	84.8 ± 10.2	92.0 ± 6.6 *	77.6 ± 7.6
FM (kg)	18.0 ± 4.6	20.9 ± 4.1 *	15.0 ± 3.0
Fat% (%)	17.3 ± 3.0	18.4 ± 2.6 *	16.3 ± 3.0
RMR (kcal·day^−1^)	2585 ± 176	2706 ± 94 *	2465 ± 156

Mean ± SD. BM = body mass, FFM = fat-free mass, FM = fat mass, Fat% = percent body fat, RMR = resting metabolic rate. * Indicates significant difference between forwards and backs (*p* < 0.05).

**Table 3:** Correlation matrix between predictor variables and resting metabolic rate among all players (*n* = 108).

	Age	Height	BM	FFM	FM	Fat%	RMR
Age	1.00						
Height	0.17	1.00					
BM	0.18	0.56 **	1.00				
FFM	0.24 *	0.61 **	0.96 **	1.00			
FM	−0.00	0.30 **	0.79 **	0.59 **	1.00		
Fat%	−0.14 *	0.04	0.40 **	0.12	0.87 **	1.00	
RMR	0.14	0.54 **	0.81 **	0.84 **	0.49 **	0.10	1.00

BM = body mass, FFM = fat-free mass, FM = fat mass, Fat% = body fat percentage, RMR = resting metabolic rate. * Correlation is significant at *p* < 0.05. ** Correlation is significant at *p* < 0.01.

**Table 4:** Comparisons between measured resting metabolic rate in the current study (*n* = 108 professional male rugby union players) and resting metabolic rates derived from the prediction equations.

RMR Method	RMR (kcal·day^−1^)	Mean Difference (mean ± SD)	95% CI of MD	Effect Size (*d*)	Sig (*p*)	RMSE (kcal·day^−1^)
*Measured (IC)*	2584 ± 176					
Harris–Benedict [28]	2238 ± 201	−346 ± 120	−323 to −369	−2.89	<0.001	366
Cunningham [29]	2330 ± 211	−254 ± 133	−228 to −279	−1.91	<0.001	286
ten Haaf ^FFM^ [23]	2378 ± 219	−206 ± 137	−179 to −232	−1.50	<0.001	247
ten Haaf ^BM^ [23]	2332 ± 181	−252 ± 113	−230 to −273	−2.23	<0.001	276
Jagim [30]	2778 ± 259	194 ± 157	164 to 224	1.23	<0.001	249
Mackenzie-Shalders ^LBM^ [15]	2447 ± 285	−137 ± 185	−102 to −172	−0.74	<0.001	229
Mackenzie-Shalders ^BM^ [15]	2417 ± 213	−167 ± 126	−143 to −191	−1.32	<0.001	209
Tinsley ^FFM^ [14]	2480 ± 263	−104 ± 150	−75 to −132	−0.69	<0.001	182
Tinsley ^BM^ [14]	2562 ± 331	−22 ± 216	19 to −63	−0.10	0.295	216
*Newly Developed Equations*						
Posthumus ^FFM^	2585 ± 146	1.6 ± 96	−20 to 17	0.02	0.861	96
Posthumus ^BM^	2585 ± 140	1.6 ± 105	−22 to 18	0.02	0.875	104

Resting metabolic rate (RMR) presented as mean ± standard deviation (SD). Mean difference (MD) in RMR between measured and prediction equations presented as mean ± SD, and 95% CI of MD = 95% confidence interval of mean difference (lower bound to upper bound). IC = indirect calorimetry, FFM = fat-free mass, BM = body mass, LMB = lean body mass, Sig = significance, RMSE = root mean squared error. Significance = *p* < 0.05.

## Data Availability

The data presented in this study are available upon reasonable request from the corresponding author and the permission of all parties involved in this study.
